# Antisense oligonucleotides directed against *App* and *Rab5* normalized endosomal Rab activity and reversed DS‐AD‐linked degenerative phenotypes in the Dp16 mouse model of Down syndrome

**DOI:** 10.1002/alz.70022

**Published:** 2025-05-07

**Authors:** Xu‐Qiao Chen, Xinxin Zuo, Ann Becker, Michael Mante, Jazmin B. Florio, Satish G. Jadhav, Ricardo Albay, Aaron Johnstone, Dmitry Karachentsev, Robert Rissman, Hien Zhao, Steven F. Dowdy, William C. Mobley

**Affiliations:** ^1^ Department of Neurosciences University of California San Diego La Jolla California USA; ^2^ Department of Cellular & Molecular Medicine University of California San Diego La Jolla California USA; ^3^ Ionis Pharmaceuticals Inc. Carlsbad California USA

**Keywords:** Alzheimer's disease, *App*‐ASO, Down syndrome, Dp16 mouse, endolysosomal network, neurotrophin signaling, *Rab5*‐ASO, synaptic proteins, tau phosphorylation

## Abstract

**INTRODUCTION:**

Down syndrome (DS) markedly raises the risk of Alzheimer's disease (DS‐AD). Our findings identified widespread dysregulation of the endolysosomal network (ELN) in DS and DS‐AD brains, driven by increased *APP* gene dose, hyperactivation of RAB5, and elevated levels of guanine nucleotide exchange factors (GEFs) for RABs 7 and 11.

**METHODS:**

We investigated whether increasing *APP* gene dose and RAB5 hyperactivation contributed to neuropathogenesis and whether a clinically feasible intervention could reverse ELN changes. The Dp16 DS‐AD mouse model was treated with a mouse *App*‐specific antisense oligonucleotide (*App*‐ASO) and *Rab5*‐specific ASOs targeting *Rab5a* and *Rab5b*.

**RESULTS:**

*App*‐ASO treatment normalized full‐length APP (fl‐APP) and its products, RAB5 activity, and downstream RABs 7 and 11 pathways. *Rab5*‐ASOs reduced RAB5 levels and restored endosomal Rab activity. Both ASO treatments mitigated DS‐AD‐linked pathologies.

**DISCUSSION:**

These findings highlight ELN dysregulation in DS and the therapeutic potential of ASO‐based strategies targeting *APP* or *Rab5* to counteract DS‐AD features.

**Highlights:**

- *App*‐ASO treatment reduced the levels of APP and its products and normalized endosomal Rab activity and GEF levels in Dp16 mice.
- Administration of *Rab5*‐ASOs reduced RAB5 levels and normalized endosomal Rab activity and GEF levels in Dp16 mice.
- Both ASO treatments were well tolerated and mitigated APP‐linked pathologies including tau hyperphosphorylation, neurotrophin signaling deficits, and synaptic protein loss.
- *App*‐ASO or *Rab5*‐ASOs reversed established pathological phenotypes in Dp16 mice.

## 1 BACKGROUND

Alzheimer's disease (AD) is the most common form of dementia, yet its underlying mechanisms remain incompletely understood. Down syndrome (DS), caused by an extra partial or complete copy of chromosome 21, is the most prevalent genetic form of AD.^1^, ^2^, ^3^ Apart from rare cases of autosomal‐dominant AD, no causative gene mutation is found in most people with AD. However, there is compelling evidence for a necessary role for increased gene dose for the amyloid precursor protein (*APP)* in those with DS diagnosed with AD (DS‐AD).^4^, ^5^ Targeting expression of the gene for *APP* has thus emerged as a key target for therapies to combat DS‐AD.

The full‐length APP (fl‐APP) protein is processed to produce various amyloid beta (Aβ) species; Aβ40 and Aβ42 are the predominant components of the amyloid plaques found in AD.^6^ This hallmark of AD pathology, and the Aβ‐induced hyperphosphorylation of tau that constitutes the principal component of the neurofibrillary tangle that serves as the second classical hallmark, are linked to neurodegeneration and cognitive decline.^7^ Recently, several monoclonal antibodies targeting Aβ have shown promise in treating early‐stage AD, promoting clearance of plaques, reducing markers of tau pathology, and modestly slowing disease progression. While encouraging, anti‐Aβ antibodies have demonstrated a significant incidence of amyloid‐related imaging abnormalities (ARIA), pointing to this adverse effect as potentially limiting utility. Given the prominence of amyloid pathology involving blood vessels in the DS brain – that is, congophilic angiopathy – concern for their safe use in this population is appropriate. Accordingly, trials to assess efficacy and safety in those with DS are reportedly forthcoming.

An alternative therapeutic strategy involves directly targeting *APP* gene expression, at either the transcriptional or translational level. As one example, in studies in the Ts65Dn mouse model of DS, we administered Posiphen, a small molecule that inhibits the translation of *App* mRNA. This treatment normalized levels of fl‐APP and its downstream products, reversed RAB5 hyperactivation, rescued neurotrophin signaling deficits, and reduced tau hyperphosphorylation.^8^ One potential drawback is that Posiphen may inhibit the translation of other proteins’ mRNAs,^9^ thereby raising concerns about potential off‐target effects. Another approach, as practiced successfully to treat other neurodegenerative disorders, would employ antisense oligonucleotides (ASOs) to target *APP* expression selectively.^10^, ^11^ *APP*‐specific antisense oligonucleotides (*APP*‐ASOs) have been applied to in vitro induced pluripotent stem cell (iPSC)‐derived neurons from individuals with DS or AD with *APP* duplications. *APP*‐ASOs corrected endolysosomal network (ELN) dysfunction.^12^ However, whether or not this approach can be applied safely and effectively in vivo is yet to be addressed. Nor is it clear whether or not and to what extent this approach would mitigate or reverse AD‐associated neuropathologies in the brain.

Increased *APP* gene dosage is linked to several AD‐related pathologies, including early endosome enlargement, disrupted neurotrophin signaling and axonal transport, reduced retromer complex and synaptic proteins, tau hyperphosphorylation, microgliosis, astrocytosis, and lysosomal dysfunction.^8^, ^13^, ^14^, ^15^, ^16^, ^17^, ^18^ We recently reported widespread ELN disruption in both DS and DS‐AD brains, driven by APP overexpression via its β‐CTF, leading to RAB5 hyperactivation.^19^ In DS models, RAB5 hyperactivation increased the levels and endosomal membrane binding of the guanine nucleotide exchange factors (GEFs) for endosomal RABs 7 and 11, disrupting the ratio of their GEFs to GTPase binding proteins (GAPs). This imbalance impaired endosomal maturation, tightly regulated by GTPases.^20^, ^21^ GEFs activate specific Rabs for effector binding, while GAPs inactivate them.^20^ For this reason, reducing RAB5 activity emerges as a potential DS therapy. Consistent with this, inhibition of p38α, a regulator of RAB5, reverses RAB5‐mediated endosomal enlargement and restores basal forebrain cholinergic neurons (BFCNs) in a DS mouse model.^22^ However, p38 regulates processes beyond the ELN.^23^ Thus, an ASO strategy targeting *Rab5* offers a precise approach to modulating its activity.

In the current study carried out in the Dp16 mouse model of DS, in vivo administration of *App*‐ and *Rab5*‐specific ASOs reduced *App* and *Rab5* gene expression, normalized RAB5 activity, and restored other endosomal Rab activities. Remarkably, both ASOs reversed neurotrophin signaling deficits, synaptic protein loss, and abnormal tau phosphorylation. These findings further support that increased *APP* expression and RAB5 hyperactivation contribute to ELN dysregulation and AD‐ and DS‐AD‐related phenotypes. Targeting *APP* and/or *Rab5* with ASO treatments shows promise for preventing or reversing neurodegeneration in DS‐AD.

## 2 METHODS

### 2.1 Ethical approval

All animal studies were conducted in strict accordance with the guidelines outlined in the Guide for the Care and Use of Laboratory Animals by the National Institutes of Health. The experiments were approved by the University of California San Diego Institutional Animal Care and Use Committee (Protocol No.: S09315).

### 2.2 Reagents

Halt Protease and Phosphatase Inhibitor Cocktail (78440), poly‐D‐lysine hydrobromide (PDL; P6407), transferrin (Tf)‐biotin (T3915), horseradish peroxidase (HRP)‐conjugated streptavidin (N100), EZ‐Link Sulfo‐NHS‐LC‐LC‐Biotin (21338), acetonitrile (AX0151), and methylene blue (M9140) were obtained from MilliporeSigma. HBSS (14185052), trypsin (2.5%, 10×) (15090046), neurobasal medium (21103049), B‐27 supplement (17504044), GlutaMAX supplement (35050061), Pierce NeutrAvidin Agarose (29200), brain‐derived neurotrophic factor (BDNF, RP‐8642), 4′,6‐diamidino‐2‐phenylindole (DAPI, 62248), Fluoromount‐G Mounting Medium (00‐4958‐02), and LysoTracker™ Red DND‐99 (L7528) were obtained from Thermo Fisher Scientific. DNAase I (10104159001) was obtained from Roche. Fetal bovine serum (FBS; FB‐02) was obtained from Omega Scientific. BMV109 was a gift from Dr. Matthew Bogyo (Stanford University School of Medicine). Acetonitrile (AX0151), 3‐((Dimethylamino‐methylidene)amino)‐3H‐1,2,4‐dithiazole‐3‐thione (DDTT, 40‐4137‐52), and triethylamine acetate (TEAA, 60‐4110‐60) were obtained from Glen Research. Acrylamide (161‐0144) and the Iscript cDNA Synthesis Kit (1708891) were obtained from Bio‐Rad. Urea (AC327380050) was obtained from Thermo Fisher Scientific. The VPLEX Aβ Peptide Panel 1 (4G8) Kit (K15199E) was obtained from Meso Scale Discovery (MSD). The Quick‐RNA Miniprep Kit (R1054) was obtained from Zymo Research.

### 2.3 Mice

Dp (16)1Yey/+ (Dp16; JAX:013530; Jackson Laboratory) mice harbor a duplication orthologous to the human chromosome 21 (HSA21) q11‐q22.3 and carry 113 genes homologous to those on HSA21. To maintain the Dp16 mice, females were crossed with (C57BL/6J [JAX:000664; Jackson Laboratory] × C3H/HeJ [JAX:000659; The Jackson Laboratory]) F1 mice (B6C3). Diploid (2N) littermate mice, with identical genetic backgrounds, were utilized as controls. The animals' genotype was confirmed through PCR using genomic DNA extracted from tail samples. The PCR protocol included amplification of the *HPRT* insertion specific to Dp16 mice, alongside amplification of the *IL‐2* gene serving as an internal control. The primer sequences used for *HPRT* were as follows: forward: 5′‐AGGATGTGATACGTGGAAGA; reverse: 5′‐CCAGTTTCACTAATGACACA; while the primers for *IL‐2* were as follows: forward: 5′‐CTAGGCCACAGAATTGAAAGATCT; reverse: 5′‐GTAGGTGGAAATTCTAGCATCATCC. C57BL/6J mice were crossed to produce WT embryos. All animals were bred and maintained following standard procedures, housed two to five per cage with a 12‐h light‐dark cycle, and provided ad libitum access to food and water. Only male mice were used for the in vivo study.

**RESEARCH IN CONTEXT**

1. **Systematic review**: DS significantly increases the risk of AD. Our research revealed widespread dysregulation in the endolysosomal network in DS and DS‐AD brains with changes driven by an elevated *APP* gene dose and RAB5 hyperactivation.
2. **Interpretation**: Our findings position RAB5 as a central hub in endolysosomal dysfunction and link these changes to the neurodegenerative processes observed in DS‐AD.
3. **Future directions**: Modulating *APP* or *Rab5* gene expression may prove to be a clinically feasible approach to preventing or reversing DS‐AD‐related neurodegeneration. Future studies should explore and refine ASO‐based therapies and evaluate their long‐term safety and efficacy in clinical applications in DS and DS‐AD.

### 2.4 ASO synthesis

The sequences of the ASOs utilized in this study are provided in Table S1. The *App*‐ASO was a gift from Ionis Pharmaceuticals. For the *Rab5*‐ASO, Integrated DNA Technologies (Coralville, IA) designed several hits targeting *Rab5a* or *Rab5b*. For each one, 15 sequences were selected and screened in primary cortical neurons dose‐dependently. Ultimately, one *Rab5a*‐ASO and two *Rab5b*‐ASOs were identified for their efficient knockdown effects, which were then utilized for large‐scale synthesis. 20mer *Rab5a* and *Rab5b* gapmer ASOs were synthesized on a Mermade‐6 oligonucleotide synthesizer (Bioautomation) at 1 µmole scale using standard Q‐T columns (Glen Research). Commercially available phosphoramidites were resuspended in acetonitrile to 0.1 M and coupled per the manufacturer's recommendation (R.I. Chemicals; Glen Research). All C positions contained a 5‐Me base modification. All RNA positions (1‐5, 16‐20) contained a 2′‐MOE (methoxyethyl) modification, and all DNA positions (6‐15) contained a 2′‐H. All phosphate linkers were sulfurized with 3‐((N, N‐dimethyl‐aminomethylidene) amino)‐3H‐1,2,4‐dithiazole‐5‐thione) to yield a full phosphorothioate oligonucleotide. Oligonucleotides were deprotected in ammonia for 18 to 24 h at 55°C, evaporated, and purified by RP‐HPLC (1200 Series Analytical, Agilent) on a Zorbax SB‐C18 column (Agilent) using a linear acetonitrile gradient with 50 mM TEAA (60‐4110‐60; Glen Research) counterion. Oligonucleotides were analyzed by matrix‐assisted laser desorption ionization–time of flight (MALDI‐TOF) mass spectrometry using a THAP matrix on a DE‐Pro MALDI‐TOF mass spectrometer (Voyager, Applied Biosystems) and by 15% acrylamide/7 M urea denaturing gel electrophoresis stained with methylene blue (M9140; MilliporeSigma). Oligonucleotides were stored lyophilized at −80°C.

### 2.5 Intracerebroventricular (ICV) injection of ASO

Surgical instruments were sterilized before use. Isoflurane was administered via a vaporizer set to 3% for anesthesia, while the infusion pump delivered at a rate of 1 µL/min. Anesthesia induction was carried out in a chamber, with confirmation of effectiveness through a foot‐pinch response. Once anesthetized, the mouse was positioned in the stereotaxic apparatus, ensuring the correct placement of the nose cone for continuous isoflurane flow. Stability was ensured by gently pressing on the mouse's head. Buprenorphine (0.05 mg/kg) was administered subcutaneously for analgesia. During the surgery, a heating pad was placed beneath the mouse to prevent hypothermia, and ophthalmic lubricant was applied to the eyes to prevent corneal dryness. The surgical area was prepared by shaving the fur and cleaning it with alternating applications of 70% ethanol and povidone‐iodine. An incision approximately 10 to 15 mm in length was made down the center of the head, exposing the skull. Using a digital stereotaxic apparatus, the coordinates for the ICV injection site were determined (+ 0.3 mm anteroposterior, + 1.0 mm mediolateral, −3.0 mm dorsoventral). A skin marking pen was used to mark the desired position. A small hole was carefully drilled at the marked location, ensuring no damage was caused to the brain. The syringe, mounted with a 10 µL Hamilton syringe (80366 26 s ga; Hamilton), was positioned at the bregma, and the coordinates were zeroed. The needle was slowly lowered to the target depth of −3.0 mm, and the ASOs (Table S1) or vehicle (phosphate‐buffered saline [PBS]) were administered in a volume of 5 µL at a rate of 1 µL/min using the infusion pump. Following infusion, a 1‐min wait period was observed before slowly retracting the needle. The incision was closed using an approved skin glue (GLUture; World Precision Instruments). The mouse was then removed from the stereotaxic apparatus and monitored until fully recovered. The behavior of the treated mice was monitored daily until sacrifice. We observed no obvious difference in weight gain or activity for ASO‐treated mice.

### 2.6 Determination of *App*‐ASO distribution in mouse brain

The *App*‐ASO‐treated mouse brain was drop‐fixed in ice‐cold 4% paraformaldehyde (PFA) in PBS overnight with rocking, then sequentially cryopreserved in 20% sucrose, followed by 30% sucrose in PBS overnight at 4°C. Brain sections were collected using a sliding microtome at a thickness of 40 µm. To determine the distribution of ASO, sections underwent immunohistochemistry using an anti‐ASO antibody. Floating sections were permeabilized and blocked in PBS containing 3% FBS and 0.3% Triton X‐100 for 15 min at room temperature. They were then incubated with an anti‐ASO antibody (1:1000) in the same buffer overnight at 4°C. Following this, sections were washed three times in PBS for 15 min each and subsequently incubated for 1 h at room temperature with PBS containing the anti‐rabbit Alexa Fluor 555 secondary antibody (1:500). After washing three times in PBS for 15 min each, sections were counterstained with DAPI before mounting with Fluoromount‐G. The sections were imaged using a Leica DMI8 widefield microscope and LAS X software.

### 2.7 RNA isolation and quantitative PCR

Total RNA was extracted from the cortex and hippocampus of 2N and Dp16 mice using the Quick‐RNA Miniprep Kit. Equivalent quantities of RNA were then used to synthesize cDNA with the iScript™ cDNA Synthesis Kit, adhering to the manufacturer's protocol. The primer sequences are provided below (Table S2). qPCR was conducted for 40 cycles. Values within the log‐linear phase of the amplification curve were determined for each probe/primer set and analyzed using the ΔΔCt method (Applied Biosystems 7300 Real‐Time PCR System).

### 2.8 Western blotting

Equal amounts of total protein (10 to 20 µg) from each sample were separated by SDS‐PAGE and then transferred to PVDF membranes. The membranes were blocked with 5% non‐fat milk for 1 h and incubated overnight at 4°C with specific primary antibodies (Table S3). Afterward, the membranes were incubated with goat anti‐rabbit IgG‐HRP (1:15,000) or anti‐mouse IgG‐HRP (1:15,000) at room temperature for 1 h. Blots were developed using BioRad Clarity Western ECL substrate and imaged with the ChemiDoc XRS+ (Bio‐Rad). Only blots with signals within the linear range were quantified using ImageLab 3.0.1 software (Bio‐Rad).

### 2.9 Measurements of Aβ40 and Aβ42 with MSD assay

Concentrations of Aβ40 and Aβ42 were measured using the VPLEX Aβ Peptide Panel following the manufacturer's instructions. The cortices of 2N and Dp16 mice after either *App*‐ASO or *Rab5*‐ASO treatment were dissected and homogenized in radioimmunoprecipitation assay (RIPA) buffer (25 mM Tris‐HCl pH 7.5, 150 mM NaCl, 1% NP‐40, 1% sodium deoxycholate, 0.1% SDS) containing Halt protease and phosphatase inhibitors using the Bullet Blender (Next Advance). The protein concentrations of all brain lysates were adjusted to 10 mg/ml with the same lysis buffer before measurement. Data were obtained using the MESO QuickPlex SQ 120 and analyzed using Discovery Workbench 3.0 (Meso Scale Diagnostics).

### 2.10 Guanosine‐5′‐triphosphate (GTP) agarose pull‐down assay

Dissected mouse brain tissues were homogenized in lysis buffer (50 mM Tris‐HCl pH 7.5, 250 mM NaCl, 5 mM Mg‐Acetate, 0.5% Triton X‐100, protease inhibitor cocktail, and 0.2 mM sodium orthovanadate) using 10 to 15 strokes in a 2‐mL Wheaton Dounce homogenizer (DWK Life Sciences) and then rotated at 4°C for 30 min. All lysates were centrifuged at 12,000 rpm for 15 min at 4°C to isolate the supernatants. Protein concentration in the supernatants from the mice was determined using the Bradford assay. An aliquot of the supernatant was reserved for measuring the total levels of individual Rab proteins. Equal amounts of supernatants were incubated with 100 to 150 µL GTP‐agarose beads overnight at 4°C with rotation. Each sample contained 1 mg total protein. The beads were washed three times (5 min each) at 4°C with lysis buffer lacking protease inhibitors. After washing, the beads were boiled in SDS‐PAGE sample buffer. GTP‐bound Rab proteins were quantified by Western blotting.

### 2.11 Primary neuron culture

E18 WT mouse embryos and embryos from 2N or Dp16 mice were used to generate cortical neurons for cultures following established protocols.^8^ Genomic DNA for genotyping was extracted from tail samples of each embryo using the procedure outlined previously. Cortical tissue was carefully dissected from the embryos, then finely minced with a sterile, fire‐polished Pasteur glass pipette, followed by gentle trituration. The tissue was further dissociated by incubating in 1× trypsin in HBSS at 37°C for 5 min, then treated with DNAase I (1 mg/ml). After dissociation, the tissue was triturated again to obtain single cells. The dissociated cells were neutralized using Neurobasal medium containing 10% FBS, B‐27, and GlutaMAX and then centrifuged briefly at 1000 rpm for 5 min. The resulting cell pellet was resuspended and plated in PDL‐coated dishes with Neurobasal medium supplemented with 10% FBS, B‐27, and GlutaMAX, and incubated overnight. The following day, the medium was replaced with maintenance medium (Neurobasal with B‐27 and GlutaMAX), and half of the medium was refreshed every 2 to 3 days until the experiments were performed.

### 2.12 ASO treatment of primary cortical neurons

The optimal concentration of *App*‐ASO was determined in our pilot studies, where a 100 nM treatment for 4 days effectively reduced APP protein levels in Dp16 primary neurons to levels comparable to those in 2N neurons. For subsequent experiments, primary Dp16 cortical neurons were cultured until DIV10 and then treated with 100 nM *App*‐ASO for 4 days before proceeding with further measurements as indicated.

To evaluate the efficacy of *Rab5*‐ASOs in vitro, WT mouse cortical neurons at DIV10 were treated with various *Rab5*‐ASO candidates targeting either *Rab5a* or *Rab5b* at different concentrations, as specified. After 4 days of treatment, cells were harvested to assess the knockdown efficiency of each ASO.

### 2.13 Cathepsin activity measurement with BMV‐109

Mouse cortical neurons were cultured on PDL‐coated 12‐well plates for 2 weeks. To monitor cathepsin activity, the pan‐cysteine cathepsin activity‐based probe BMV‐109 – targeting cathepsins X, B, S, and L^24^ – was diluted in neuronal culture medium to a final concentration of 1 µM. The probe was incubated with the neurons at 37°C for 45 min. After incubation, the medium was gently aspirated, and the cells were washed with PBS. The cells were subsequently harvested and lysed using 1 × SDS loading buffer. The lysates were then resolved on a SDS‐PAGE gel, alongside a fluorescent molecular weight marker. The gel was imaged using a Typhoon Imager with Cy5‐optimized excitation and emission settings. After imaging, the proteins were transferred onto a PVDF membrane for Western blot analysis.

### 2.14 LysoTracker

Cortical neurons isolated from 2N and Dp16 mouse embryo brains were seeded in 35‐mm glass‐bottom dishes coated with PDL and treated as indicated. After 2 weeks’ culture, the culture was replaced with prewarmed medium containing 50 nM LysoTracker™ Red DND‐99 and incubated at 37°C for 30 min. After washing with fresh culture medium, live cell images were acquired with an Olympus FluoView FV1000 confocal microscope with a 60 × NA 1.20 water immersion objective. ImageJ software was used to process and analyze the basic images.

### 2.15 Constructs

To generate lenti‐RAB5^S34N^ for neuronal studies, we utilized the Lox‐Syn lentiviral vector, which features two neuron‐specific synapsin promoters. One promoter drives the expression of the target protein, while the other controls green fluorescent protein (GFP) expression, enabling visualization.^25^ A control vector was similarly constructed by omitting DsRed while preserving GFP functionality in the Lox‐Syn backbone. Additionally, the helper plasmids pMD2.G (Addgene No. 12259) and psPAX2 (Addgene No. 12260) were obtained from Addgene to support lentiviral production.

### 2.16 Lentivirus production and transduction

Lentiviral particles were generated in HEK293T cells via co‐transfection with expression vectors and the viral packaging plasmids pMD2.G and psPAX2 using the calcium phosphate transfection method. Following production, the viral particles were concentrated by ultracentrifugation at 24,000 rpm for 2 h using an SW‐28 rotor. The concentrated viruses were then resuspended in Ca^2^⁺/Mg^2^⁺‐free PBS and applied to neurons.

### 2.17 Tf uptake assay

Cultured 2N or Dp16 cortical neurons were prepared in a 24‐well plate (3.5 × 10^5^ neurons each well) and transduced with lenti‐control or lenti‐RAB5^S34N^ from DIV5. After 72 h, the cells were starved for 2 h in Neurobasal medium before being exposed to 10 µg/ml of biotin‐coupled transferrin (tf‐biotin) for the specified durations. To remove surface‐bound biotin‐labeled Tf, the cells were treated with glycine buffer (100 mM NaCl, 50 mM glycine, pH 3), followed by two washes with cold PBS before lysing in buffer (50 mM Tris‐HCl pH 7.5, 150 mM NaCl, 1% Triton X‐100, Halt Protease and Phosphatase Inhibitor Cocktail) for Western blotting analysis. Following electrotransfer, PVDF membranes were incubated with HRP‐conjugated streptavidin (1:100,000) at room temperature for 1 h.

### 2.18 Surface biotinylation

Primary 2N and Dp16 cortical neurons in 60‐mm dishes (3.5 × 10^6^ neurons each well) transduced with lenti‐RAB5^S34N^ or lenti‐control were biotinylated using 0.25 mg/ml Sulfo‐NHS‐LC‐biotin in PBS containing Mg^2+^/Ca^2+^ for 45 min at 4°C, followed by quenching with glycine for 20 min. The cells were lysed in RIPA buffer (50 mM Tris‐HCl pH 7.5, 150 mM NaCl, 1% Triton X‐100, 1 mM PMSF, 1 mg/ml aprotinin, leupeptin, and pepstatin), and the lysates were incubated with NeutrAvidin beads overnight at 4°C. The pull‐down samples and 5% of total lysates were processed for immunoblotting.

### 2.19 BDNF‐induced signaling transduction in primary neurons

Mass cultures of either 2N or Dp16 cortical neurons in a 24‐well plate (3.5 × 10^5^ neurons each well), transduced with lenti‐control or lenti‐RAB5^S34N^ from DIV5 for 72 h, were starved for 2 h in Neurobasal medium before stimulation with 20 ng/ml BDNF (RP‐8642; Thermo Fisher Scientific) for different durations. The neurons were then lysed in buffer (50 mM Tris‐HCl pH 7.5, 150 mM NaCl, 1% Triton X‐100, Halt Protease and Phosphatase Inhibitor Cocktail), followed by centrifugation (12,000 rpm for 15 min at 4°C) to produce supernatants for Western blotting. This was performed to examine the activation of tropomyosin receptor kinase B (TRKB), protein kinase B (AKT), and extracellular signal‐regulated kinase (ERK).

### 2.20 Statistic analysis

All data are presented as the mean ± SEM. Statistical analyses were performed using PRISM with a Student's *t*‐test, or one‐way analysis of variance (ANOVA) test followed by the Newman–Keuls multiple‐comparisons test. The significance levels were *p < 0.05, **p < 0.01, and ***p < 0.001.

## 3 RESULTS

### 3.1 *App*‐ASO reversed endosomal Rab hyperactivation and normalized RABs 7 and 11 GEF levels in brains of Dp16 mice

**FIGURE 1:**
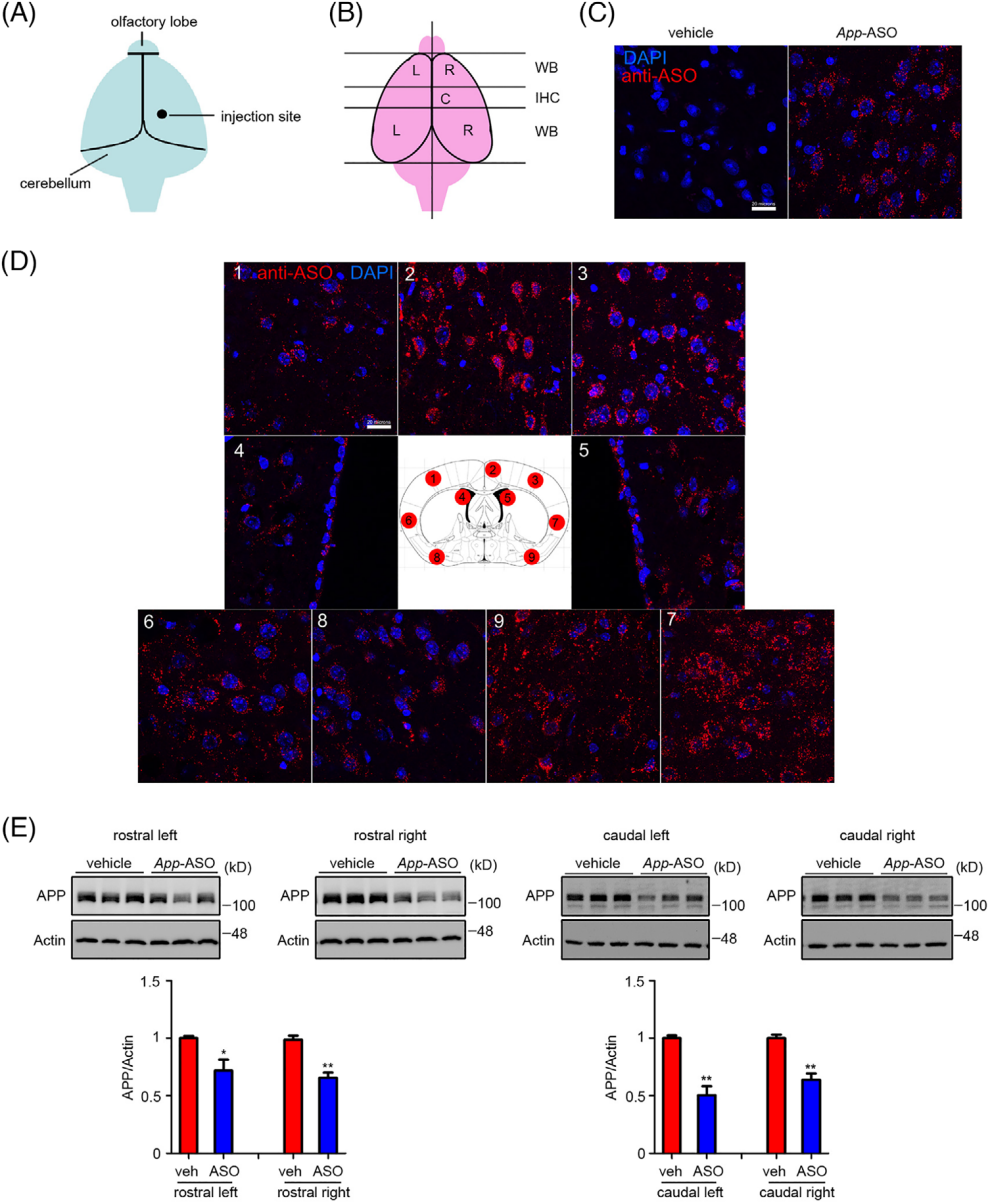
Distribution and efficiency of *App*‐ASO in mouse brains. (A) Male 2N mice, 7 to 8 months old, were treated once via ICV injection with *App*‐ASO (100 µg) or vehicle‐top view of ICV injection site in right hemisphere of 2N mouse brain. (B) The brain was dissected into five parts for biochemistry and immunohistochemistry assays. (C) Immunohistochemical staining of brain slices from central parts of brains from vehicle and *App*‐ASO‐treated mice with ASO backbone‐specific antibody. DAPI was used to label nuclei. (D) Staining showing *App*‐ASO distribution across brain's coronal section in middle. (E) Western blot analysis of the other four parts of the brain to explore the efficiency of *App*‐ASO treatment reflected by changes in APP protein levels. β‐Actin was used as loading control. Scale bar = 20 µm. Unpaired Student *t*‐test for E; *n* = 3; *p < 0.05, **p < 0.01. ASO, antisense oligonucleotide; ICV, intracerebroventricular.

Genetically reducing *App* gene dose in the Dp16 mouse prevented hyperactivation of RAB5 and downstream endosomal Rabs.^19^ To investigate whether a treatment to reduce *App* gene expression in mature Dp16 mice would have the same effect, we attempted to normalize *App* mRNA and its fl‐App product by treating 2N mice with an ASO that specifically targets *App* mRNA. To examine the distribution and efficacy of ICV‐injected ASOs, we administered 100 µg of mouse *App*‐ASO or vehicle to 2N mice at age 7 to 8 months. Two weeks later, immunostaining with an antibody targeting the ASO backbone showed widespread ASO distribution (Figure 1A–D). fl‐APP levels were significantly reduced bilaterally, with reductions of approximately 30% in the rostral region and 40% to 50% in the caudal region (Figure 1E).

**FIGURE 2:**
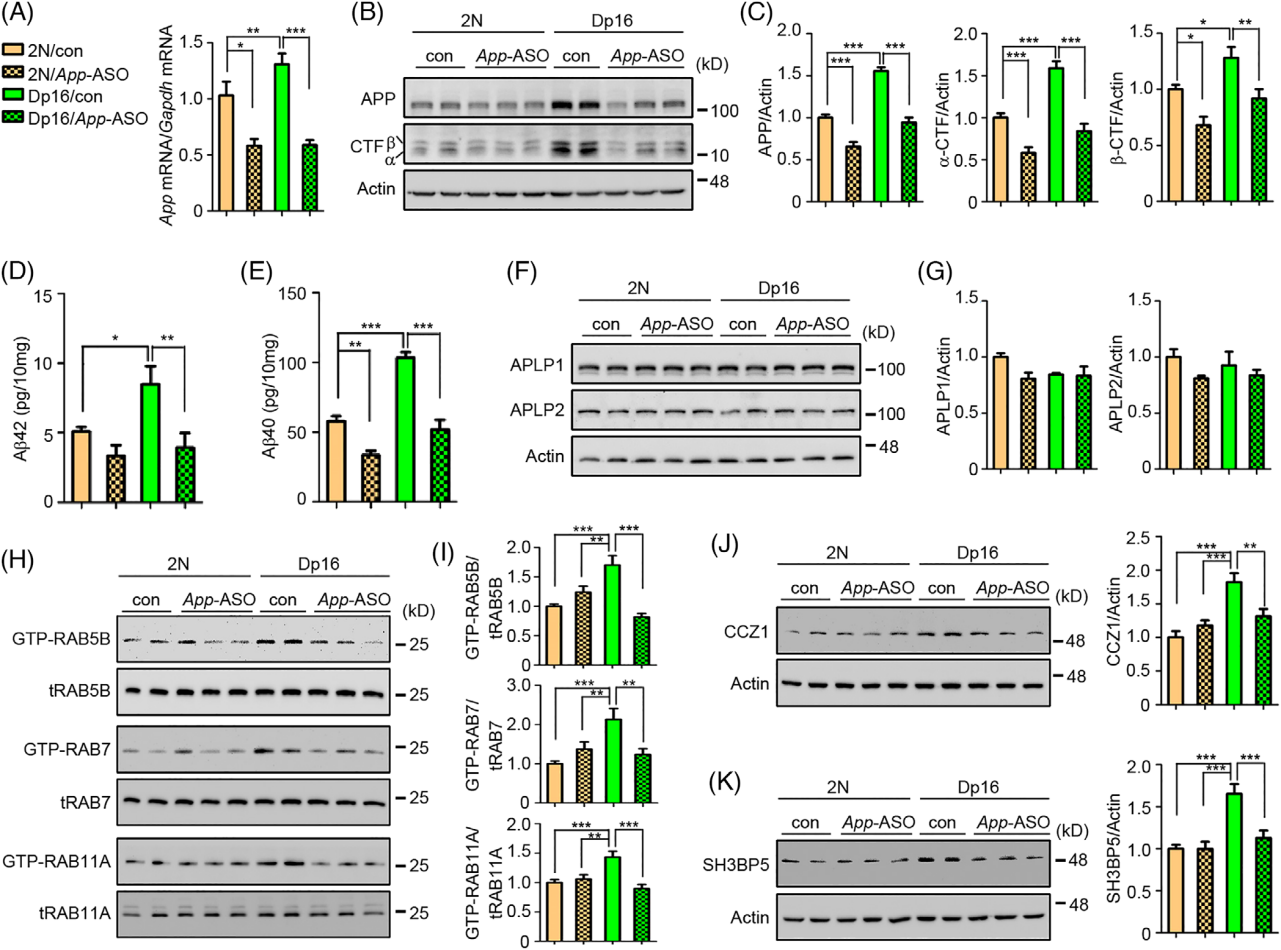
*App*‐ASO reversed endosomal Rab hyperactivation and GEF upregulation in Dp16 brains. (A) *App*‐ASO (100 µg) or vehicle was treated once via ICV injection to 6‐ to 8‐month‐old male 2N or Dp16 mice for 2 weeks followed by brain dissection. The hippocampus was used to extract mRNA and measure *App* mRNA levels by RT‐PCR, with Gapdh used as internal control. (B) Partial cortex of each mouse was processed to measure the levels of APP and CTF by Western blot, with β‐actin used as loading control. (C) Statistical analysis of APP, α‐CTF, and β‐CTF in each group was performed. (D and E) Aβ42 and Aβ40 in cortex were measured by MSD assay. (F and G) Specificity of *App*‐ASO was evaluated by examining levels of the other two APP family members APLP1 and APLP2. (H) Activities of RABs 5, 7, and 11 were measured by GTP agarose pull‐down assay in each group. (I) Statistical analysis for activities of RABs 5, 7, and 11. (J and K) Levels of CCZ1 and SH3BP5 were measured in each group. One‐way ANOVA followed by Newman–Keuls multiple‐comparisons test; *n* = 10 for C, I, J, and K, *n* = 5 for A, D, E, and G; *p < 0.05, **p < 0.01, ***p < 0.001. GTP, guanosine‐5′‐triphosphate; ICV, intracerebroventricular; MSD, Meso Scale Discovery.

Next, we tested the impact of the *App*‐ASO in 2N and Dp16 mice at 6 to 8 months of age, an age at which increases in the activities of RABs 5, 7, and 11 were present in the DS model. Two weeks after ICV injection of *App*‐ASO (100 µg) or vehicle, *App* mRNA levels in the hippocampus were evaluated. Dp16 mice carry a duplication of chromosome 16, which includes genes homologous to those on the long arm of HSA21, including the *App* gene. Consequently, Dp16 mice have three copies of the *App* gene. As expected, *App* mRNA levels in vehicle‐treated Dp16 mice were significantly elevated compared to vehicle‐treated 2N mice. The *App* mRNA levels of ASO‐injected Dp16 and 2N mice were reduced to about 60% of those in vehicle‐injected 2N mice (Figure 2A). In the cortex, fl‐APP levels in *App*‐ASO‐injected 2N mice were decreased by ∼30%; levels in Dp16 mice matched those in vehicle‐injected 2N mice. The levels of APP α‐CTF, β‐CTF, Aβ42, and Aβ40 in Dp16 cortex were also decreased to levels present in vehicle‐injected 2N mice (Figure 2B–E). A greater absolute reduction in *App* mRNA, protein, and its processing products was observed in Dp16 mice compared to 2N mice. The specificity of *App*‐ASO treatment for *App* was demonstrated by the absence of significant changes in the levels of two members of the APP family, amyloid‐precursor‐like protein 1 and 2 (APLP1, APLP2) levels (Figure 2F, G). In addition, the unchanged levels of ionized calcium‐binding adapter molecule 1 (IBA1), cluster of differentiation 68 (CD68), and mature IL‐1 beta (IL‐1β) proteins in both 2N and Dp16 brains were evidence against immune activation by *App*‐ASO treatment (Figure S1A).

Remarkably, *App*‐ASO administration normalized the activities of RABs 5, 7, and 11 in Dp16 mice, resulting in levels comparable to those in vehicle‐treated 2N controls; the treatment had no significant effect on their activities in 2N mice (Figure 2H, I). Rab activity is regulated via specific GEFs and GAPs; by loading Rabs with GTP the former activates Rabs, and GAPs inactivate Rabs by enhancing their endogenous GTPase activity.^20^ Building on our demonstration that the GEFs for RABs 7 and 11 were increased in Dp16 cortex and the necessary role played by *App* for these increases, we asked whether *App*‐ASO treatment would correct them. We found that ASO treatment significantly reduced the levels of the RABs 7 and 11 GEFs (CCZ1 and SH3BP5, respectively) in *App*‐ASO‐treated Dp16 brains, resulting in values not significantly different from those in 2N mice (Figure 2J, K). These findings are evidence that increased *App* gene expression both promotes and sustains increases in the activities of RAB5, RAB7, and RAB11 and increases in the levels of the GEFs for RAB7 and RAB11.

### 3.2 *App‐*ASOnormalized lysosomal cathepsins in Dp16 neurons

As in the DS and DS‐AD brain, the levels and activities of cathepsin B and L are increased in the Dp16 brain.^19^ To ask whether the levels of the changes in enzymes would also respond to reducing *App* gene expression, we treated primary Dp16 neurons with the *App*‐ASO. in vitro, ASO significantly reduced cathepsin B and L activity in Dp16 neurons (Figure S1B). DS fibroblasts demonstrate changes in lysosome morphology.^13^ Adding LysoTracker to the cultures revealed a marked enlargement of lysosomes in Dp16 neurons, while the lysosome number remained unchanged. Treating Dp16 neurons with the *App*‐ASO normalized lysosome size (Figure S1C). in vivo, ASO treatment markedly downregulated cathepsin L levels. Surprisingly, both cathepsins B and mature D were increased in 2N and Dp16 mice (Figure S1D–F). Why in vitro and *in vivo App*‐ASO findings differ requires additional investigation. However, when combined with the effects of *App*‐ASO on endosomal Rab activity, their impact on lysosomes demonstrates that increased *App* gene expression impacts the ELN in Dp16 brain.

### 3.3 *Rab5*‐ASOs reversed endosomal Rab hyperactivation and upregulation of RABs 7 and 11 GEFs in Dp16 brains

RAB5 hyperactivation plays a defining role in ELN dysregulation.^19^ We asked whether reducing the levels of RAB5 would counter changes in the ELN by delivering ASOs targeting *Rab5a* and *Rab5b*, isoforms highly expressed in the brain.^26^ To assess their activity, candidate *Rab5a*‐ and *Rab5b*‐ASOs were administered to WT mouse cortical neurons in vitro; dose‐dependent reductions in their respective proteins were demonstrated (Figure S2A). In studies in vivo, ICV injection of *Rab5*‐ASOs (*5a*‐1, *5b*‐1, or *5b*‐2) (100 µg each) into 3‐ to 5‐month‐old male 2N mice resulted after 2 weeks in a reduction of approximately 40% to 50% in levels of RAB5A by *Rab5a*‐1 and RAB5B by both *Rab5b1* and *Rab5b*2. *Rab5*‐ASOs did not target RAB5C. As predicted, reductions in RAB5 proteins were not accompanied by changes in the specific activity of RAB5 (GTP‐RAB5/tRAB5) (Figure S2B, 2C).

**FIGURE 3:**
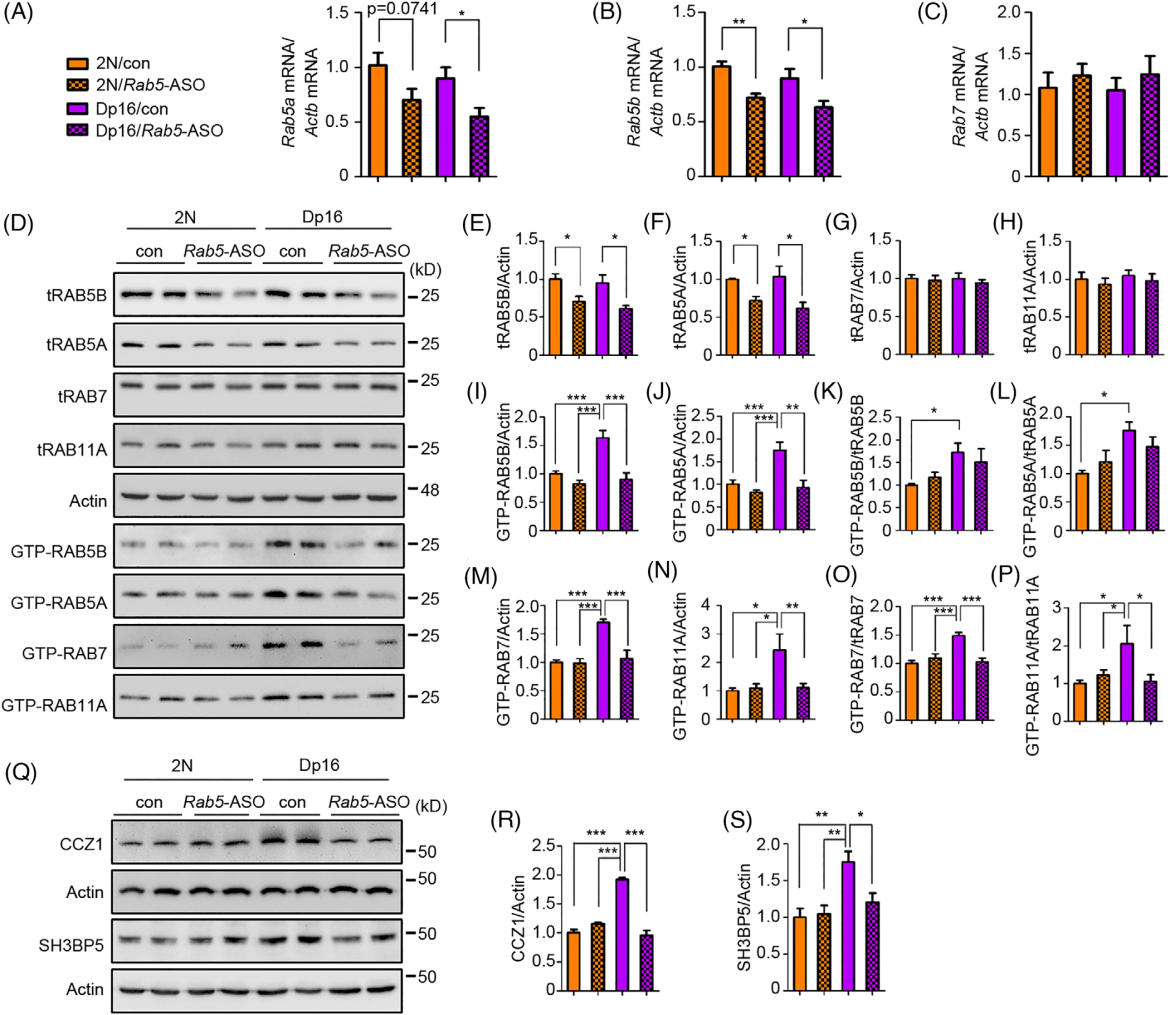
Reversal of endosomal Rab hyperactivation and GEF upregulation by *Rab5*‐ASO in Dp16 brains. (A–C) Male 2N or Dp16 mice, 9 to 10 months old were treated once via ICV injection with mixture of *Rab5a*‐ASO and *Rab5b*‐ASO (100 µg each) or vehicle. After 2 weeks, their brains were dissected. mRNA extraction was performed from hippocampus to measure mRNA levels of *Rab5a*, *Rab5b*, and *Rab7* using RT‐PCR, with *Actb* mRNA used as internal control. (D) To measure the levels of endosomal Rabs, partial cortex samples from each mouse were processed for Western blot analysis, with β‐actin used as loading control. GTP agarose pull‐down assay was used to measure the activities of RABs 5B, 5A, 7, and 11A in each group. (E–H) Quantitation and statistical analysis of levels of total Rabs in same mice. (I–P) Quantitation and statistical analysis of levels of GTP‐Rabs normalized to either actin or cognate Rabs. (Q–S) Levels of CCZ1 and SH3BP5 in each group were measured, and statistical analysis was shown. One‐way ANOVA followed by Newman–Keuls multiple‐comparisons test; *n* = 4 or 5; *p < 0.05, **p < 0.01, ***p < 0.001. GEF, guanine nucleotide exchange factor; GTP, guanosine‐5′‐triphosphate; ICV, Intracerebroventricular.

ICV co‐injection of *Rab5a*‐1 and *Rab5b*‐1 ASOs (100 µg each) in 9‐ to 10‐month‐old 2N and Dp16 mice reduced *Rab5a* and *Rab5b* mRNAs levels by ∼25% to 45% in 2N and Dp16 mice; *Rab7* mRNA was not targeted by the *Rab5*‐ASO (Figure 3A–C). No immune activation was observed, as indicated by unaltered levels of IBA1, CD68, and mature IL‐1β in both 2N and Dp16 mice treated with *Rab5*‐ASO (Figure S3A). Reductions in RAB 5A and 5B protein levels were reduced to approximately the same extent as their mRNAs; RABs 7 and 11 levels were unaffected (Figure 3D–H). The total activity of RAB 5A and 5B was normalized in Dp16 mice, with a non‐significant reduction in specific activity (Figure 3D, I–L). Importantly, both the total and specific activities of RABs 7 and 11 were normalized by *Rab5*‐ASO in Dp16 mice, with no significant impact in 2N controls (Figure 3D, M–P). Moreover, CCZ1 and SH3BP5 levels in Dp16 mice treated with *Rab5*‐ASO were reduced to levels seen in 2N controls (Figure 3Q–S). The data give further evidence that increased RAB5 activation has a necessary role in increasing RABs 7 and 11 activities and their GEF levels in the Dp16 model. They also point to ongoing hyperactivation of RAB5 as needed to sustain these changes.

### 3.4 *Rab5‐*ASO reduced the levels of CTFs in Dp16 brains

RAB5 activity mediates fl‐APP internalization.^27^ To investigate whether reducing the levels of RAB5 impacted APP processing, we evaluated fl‐APP and its products in Dp16 mice treated with *Rab5*‐ASOs. There were reductions in both α‐ and β‐CTF levels, with a slight increase in fl‐APP; given the reductions in CTFs, we were surprised to find no concomitant changes in the levels of Aβ42 and Aβ40 (Figure S3B–G). To determine whether the reductions in CTFs were due to changes in APP processing enzymes, we measured beta‐site APP cleaving enzyme 1 (BACE1), a disintegrin and metalloproteinase 10 (ADAM10), and the γ‐secretase complex levels. Although its *Bace1* mRNA levels were not changed, *Rab5*‐ASO significantly impacted BACE1 protein levels in Dp16 mice (Figure S4A, B). The mechanism and potential impact of *Rab5*‐ASO‐mediated reductions in BACE1 levels remain unclear, but this finding aligns with previous observations of p38α inhibitor effects on BACE1.^22^ Given that RAB5 mediates increases in downstream endosomal Rab activation independent of *App* gene expression,^19^ *Rab5*‐ASO may influence the activity of RABs 7 and 11 through reductions in the levels of both RAB5 and BACE1. Finally, *Rab5*‐ASO effects on cathepsin levels were similar to the *App*‐ASO, suggesting coordinated regulation of these enzymes by APP and RAB5 (Figure S4C–E).

### 3.5 *App*‐ and *Rab5*‐ASOs reversed neurotrophin signaling deficits, synaptic protein loss, and tau hyperphosphorylation

Degenerative phenotypes in DS‐AD are present together with those in the ELN. To investigate what role ELN dysregulation may contribute to degeneration, we evaluated relevant phenotypes in ASO‐treated Dp16 and 2N brains. First, we tested for other possible manifestations of RAB5 hyperactivation, which is known to increase and regulate endocytosis and endocytic trafficking.^28^, ^29^ The internalization of transferrin via the transferrin receptor (Tfr) serves as an assay for endocytosis.^30^ We observed an increase in Tf internalization in cortical neurons from Dp16 mice compared to 2N controls, an effect that was reversed by expressing a dominant negative RAB5 mutant (RAB5^S34N^) (Figure S5A). The increase in endocytosis was not due to an increase in Tfr because there was no difference in total Tfr levels (Figure S5B). To further explore the change in endocytosis, we tested for the level of Tfr on the surface of neurons. We labeled surface receptors via biotinylation and found the level to be decreased. Thus, increased endocytosis of Tf was present despite a decrease in surface levels. Significantly, as for the impact on endocytosis, expressing RAB5^S34N^ normalized surface levels (Figure S5C). These data extend the evidence for the critical role of RAB5 activity in regulating endocytosis in Dp16 neurons. The fact that this role extends beyond endocytosis is evident, as RAB5^S34N^ expression also reversed lysosome enlargement in Dp16 neurons (Figure S5D).

**FIGURE 4:**
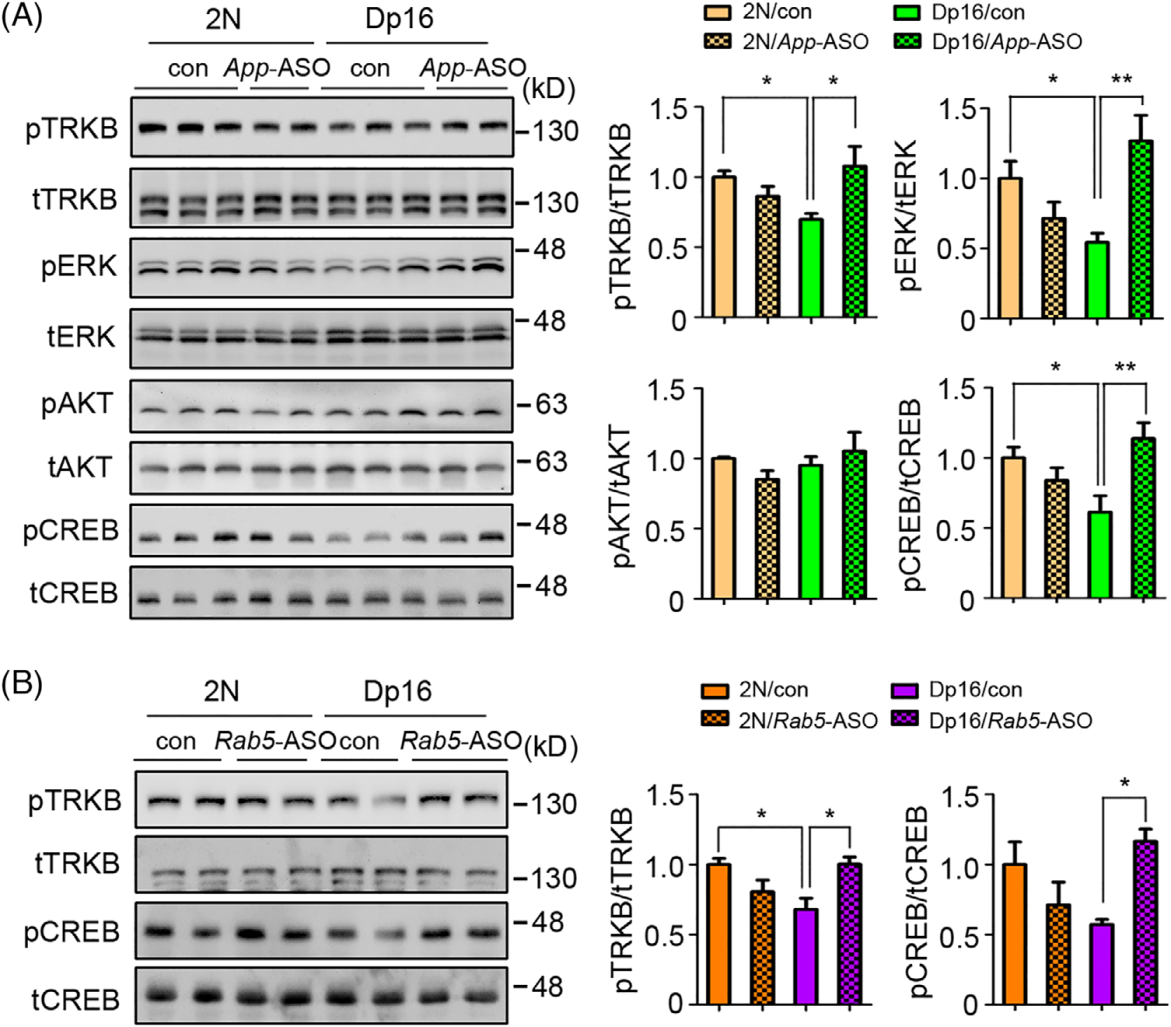
Both *App*‐ASO and *Rab5*‐ASOs recused the deficits in neurotrophin signaling in cortex of Dp16 mice. (A) The activation of TRKB, AKT, ERK, and CREB was examined in the cortex of 6‐ to 8‐month‐old male 2N and Dp16 mice treated with either *App*‐ASO or vehicle for 2 weeks. Quantitative analysis is shown on right panels. (B) The activation of TRKB and CREB was examined in the cortex of 9‐ to 10‐month‐old male 2N and Dp16 mice treated with *Rab5*‐ASO or vehicle for 2 weeks. Quantitative analysis is shown on right panels. One‐way ANOVA followed by Newman–Keuls multiple‐comparisons test; *n* = 5 to 10 for A, n = 4 or 5 for B; *p < 0.05, **p < 0.01. ASO, antisense oligonucleotides; ERK, extracellular signal‐regulated kinase; AKT: protein kinase B; TRKB, tropomyosin receptor kinase B; CREB: cyclic AMP response element binding protein.

Neurotrophin receptor signaling is regulated by the ELN.^31^ Consistent with the findings on Tfr, surface levels of TRKB, the receptor kinase receptor for BDNF, were reduced in Dp16 neurons; this change was also normalized by RAB5^S34N^ (Figure S5E), linking increased RAB5 activity to altered endocytosis of neurotrophin in Dp16 neurons. To explore the significance of changes in TRKB receptor distribution on signaling, we treated neurons in vitro with BDNF. There was reduced activation of AKT and ERK in response to BDNF in Dp16 neurons; subtle but non‐significant reductions in TRKB activation were also detected. Increased TRKB signaling through AKT was seen following Rab5^S34N^ expression, with a trend to ERK signaling also detected (Figure S5F). Remarkably, in the cortices of Dp16 mice, decreased activation of TRKB, ERK, and cyclic AMP response element binding protein (CREB) was normalized by *App*‐ASO and *Rab5*‐ASOs treatments (Figure 4A, B). These findings support a role for increased levels of fl‐APP and/or its products and increased RAB5 activation in impairing neurotrophin signaling in Dp16 neurons.

**FIGURE 5:**
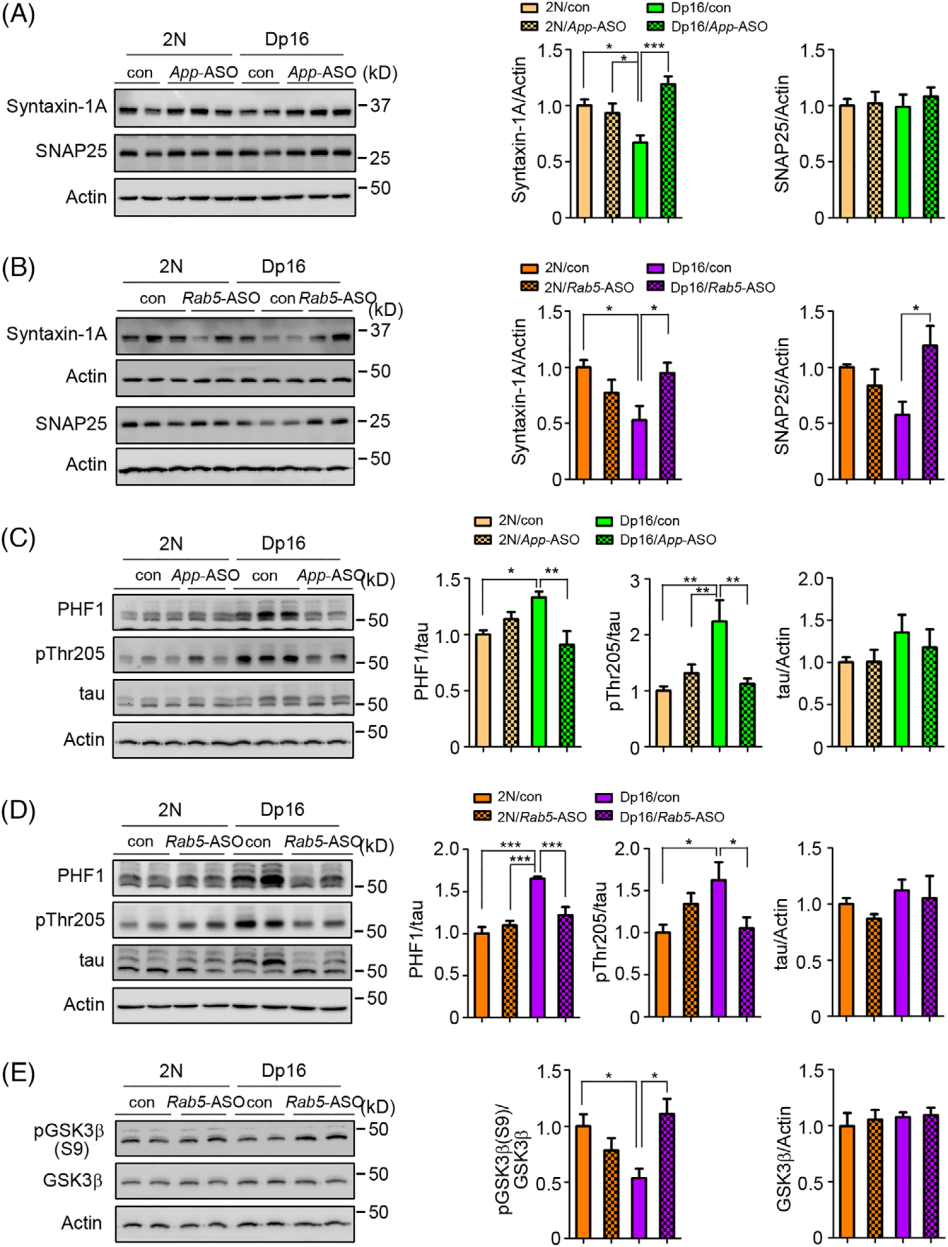
Both *App*‐ASO and *Rab5*‐ASOs reversed tau hyperphosphorylation and synaptic protein loss in the cortex of Dp16 mice. (A and B) The levels of syntaxin‐1A and SNAP25 in the cortex were measured in male 2N and Dp16 mice treated with either vehicle or *App*‐ASO (6 to 7 months old) and *Rab5*‐ASO (9 to 10 months old). (C and D) The levels of phosphorylated tau PHF1, pThr205, and total tau in the cortex of vehicle or *App*‐ASO‐ and *Rab5*‐ASO‐treated 2N and Dp16 mice were measured. (E) The activity of GSK3β in the frontal cortex of vehicle or *Rab5*‐ASO‐treated 2N and Dp16 mice was evaluated by measuring the levels of the inhibitory phosphorylation of GSK3β (Ser9). One‐way ANOVA followed by Newman–Keuls multiple‐comparisons test; *n* = 4 or 5; *p < 0.05, **p < 0.01, ***p < 0.001. ASO, antisense oligonucleotides; SNAP25, synaptosomal‐associated protein 25; GSK3β, glycogen synthase kinase‐3 beta.

Synapse dysfunction and synaptic protein loss have been proposed to play a prominent role in cognitive dysfunction in DS‐AD.^14^, ^32^ In prior studies, Dp16 mice exhibited both age‐related and *App* gene dose‐mediated reductions in the soluble N‐ethylmaleimide‐sensitive‐factor attachment protein receptor (SNARE) proteins syntaxin‐1A and synaptosomal‐associated protein 25 (SNAP25).^14^ We confirmed decreased syntaxin‐1A levels in the cortex of 6‐ to 7‐month‐old Dp16 mice and showed that this response was normalized by *App*‐ASO treatment. Reductions in syntaxin‐1A and SNAP25 observed in 9‐ to 10‐month‐old Dp16 mice were also reversed by *Rab5‐*ASOs (Figure 5A, B), underscoring roles for increased *APP* gene expression and RAB5 hyperactivation in synaptic protein maintenance.

Tau pathology is closely correlated with the onset of cognitive decline in those with DS as they convert from a cognitively stable asymptomatic state to DS‐AD.^33^ *App*‐ASO and *Rab5*‐ASOs normalized tau hyperphosphorylation in Dp16 mice, as evidenced by reduced PHF1 and pThr205 tau levels (Figure 5C, D). These treatments had minimal effects in 2N mice and did not alter total tau levels. Glycogen synthase kinase‐3 beta (GSK3β) is a tau kinase whose increased activity may contribute to aberrant tau phosphorylation in AD and DS‐AD.^34^ In Dp16 mice, reduced inhibitory pGSK3β (S9) indicated increased GSK3β activity, which was normalized by *Rab5*‐ASO treatment (Figure 5E). Consistent with earlier findings,^8^, ^35^ these data highlight increased *App* gene dose and RAB5 activity as contributors to tau hyperphosphorylation.

Taken together, the ASO studies point to the ability through selective reductions in *APP* and *Rab5* gene expression to rescue significant defects in the ELN, cellular events impacted by ELN dysregulation, and markers of DS‐AD‐linked phenotypes. These findings motivate attention to a role for ELN dysregulation in the pathogenesis of DS‐AD.

## 4 DISCUSSION

Our findings strongly support targeting *APP* and *Rab5* as therapeutic strategies to alleviate, and potentially reverse, neuronal dysfunction and degeneration in DS‐AD. A large body of evidence has demonstrated that increased *APP* gene dosage is essential for AD development in individuals with DS.^4^, ^5^ Therefore, reducing *APP* expression is a rational approach to intervention. Our recent studies also highlight the critical hub role of hyperactive RAB5, positioning it as an emerging druggable target.^19^ While several small molecules targeting different mechanisms have been developed to reduce APP protein levels or RAB5 activity,^8^, ^22^ ASOs present a particularly promising approach. Indeed, compared to other therapeutic approaches, ASOs offer the ability to target precisely the expression of specific genes and their mRNA products. In our studies, both *App*‐ and *Rab5*‐targeting ASOs were well tolerated and showed no effect on the behavior or weight of the mice (data not shown). Administration of *App*‐ and *Rab5*‐specific ASOs in the Dp16 model reduced *App* and *Rab5* gene expression, normalized RAB5 activity, restored other endosomal Rab functions, and reversed neurotrophin signaling deficits, synaptic protein loss, and abnormal tau phosphorylation. Importantly, the ASOs were highly effective even at low doses (100 µg), normalizing APP levels and RAB5 activity to those observed in 2N mice. Ideally, the use of specific, potent ASOs to reduce levels of expression of *App* or *Rab5* to levels and activities equivalent to those in the euploid condition would mitigate adverse events resulting from unwanted on‐target and off‐target effects on cellular functions. Ideally, doses would be employed that normalize the impact of *App* gene expression and RAB5 activity on all elements of the ELN, including lysosomes.

Whether or not and how changes in the ELN impact other degenerative phenotypes relevant to AD and DS‐AD is not well defined. Previous studies primarily explored this issue using overexpression models and in vitro systems. Forced overexpression of RAB5 in mice led to ELN dysregulation, neuronal dysfunction, cholinergic neurodegeneration, and impaired hippocampus‐dependent memory.^35^ β‐CTF‐induced endosomal enlargement disrupted neurotrophin signaling and impaired axonal trafficking. Additionally, overexpression of β‐CTF triggered atrophy of BFCNs through RAB5 hyperactivation in vitro.^17^ While prior studies provided valuable insights, the use of overexpression may perturb biological systems and produce findings inconsistent with normal cellular physiology. An important contribution of this study is therefore the demonstration that selective normalization of gene expression for *APP* and normalization of RAB5 activity were sufficient not only to counter changes in the ELN but also to impact changes in DS‐AD relevant phenotypes. These findings thus directly link ELN dysregulation mediated by APP and RAB5 to the pathogenesis of DS‐AD in an established in vivo model of DS. They raise the possibility that targeting the ELN may make it possible to mitigate and possibly reverse downstream pathological events. Given the utility of ASO approaches to treating neurodegenerative disorders, the findings provide direct support for this as a clinically feasible approach.

While *APP*‐ and *Rab5*‐ASOs significantly mitigated key pathological features in Dp16 mouse brains, these treatments had minimal impact on the brains of 2N mice. This finding further underscores the utility of these approaches and, equally significantly, suggests that the pathological disruption caused by increased *APP* gene dosage cannot be viewed as simply exaggerating the normal physiological processes regulated by *APP*. Understanding the mechanistic pathways through which APP and RAB5 contribute to DS‐AD pathology will be critical in refining these ASO treatments to ensure both safety and efficacy.

This study represents a pilot investigation with a treatment window of 2 weeks. However, the potential of these strategies to reverse neuronal dysfunction and loss over a longer period in the context of DS remains to be explored. The duration of efficacy of these ASOs on their targets following a single in vivo dose requires further investigation. Additionally, the optimal dosing intervals for maintaining sustained normalization of the targeted proteins need to be determined. Moreover, the treatments’ potential to mitigate cognitive and behavior‐related abnormalities over an extended period remains to be thoroughly evaluated. Currently, we are conducting a comprehensive evaluation of behavioral performance in Dp16 mice and will systematically examine the effects of these treatments on behavior. Because as yet no behavioral test in DS models has been shown to correlate with the cognitive changes in DS‐AD, research is needed to focus on this objective by developing and refining such models. Specifically, efforts should be directed toward evaluating the contributions of increased *App* gene dosage and RAB5 hyperactivation, as these may be critical for enhancing our understanding of AD‐linked dementia within the DS population.

We used only male Dp16 mice to maintain consistency, control potential biological variations, and reduce the impact of sex‐specific factors, such as hormonal fluctuations and differential gene expression patterns. However, we recognize that future studies incorporating both male and female mice are crucial to fully understand any potential sex‐specific effects of ASO treatments in the context of DS and its associated neuropathologies. Including both sexes will provide a more comprehensive view of how these treatments might affect males and females differently, potentially revealing important insights for the development of more personalized therapeutic approaches.

Targeting *APP* represents a possibly significant step toward developing precision therapies for DS‐associated dementia, potentially paving the way for more personalized and effective treatment options in this vulnerable population. Further research into the safety, efficacy, and long‐term outcomes of ASO therapies will be crucial to their success in clinical application. One important consideration is determining the optimal time to initiate treatment in individuals with DS. Given the significantly increased risk of early‐onset AD in this population, it is essential to closely monitor for early biomarkers of DS‐AD progression. Utilizing blood and imaging biomarkers to detect preclinical stages of AD (such as AD clinical stage 0) could enable earlier intervention,^36^ potentially delaying or even preventing the onset of cognitive symptoms. Excitingly, our studies also suggest that it may be possible to restore neuronal and endolysosomal function in individuals who are already affected by DS‐AD.

In summary, our study confirms the central role of RAB5 in driving endolysosomal dysfunction and extends its contribution to the neurodegenerative features characteristic of DS‐AD. Targeting *APP* or *Rab5* gene expression represents a feasible and promising approach to preventing or reversing neurodegeneration associated with DS‐AD and related conditions. Future research should focus on optimizing these ASO‐based treatments and evaluating their long‐term safety and efficacy in clinical settings.

## CONFLICT OF INTEREST STATEMENT

W.C.M. serves as a Scientific Advisory Board (SAB) member and holds stock options from Alzheon, Inc. and Promis, Inc. W.C.M. also serves as an SAB member and holds stock in Acta Pharmaceuticals, Inc. His name is on a patent under University of California San Diego and Massachusetts General Hospital concerning γ‐secretase modulators licensed to Acta Pharmaceuticals, Inc. He has served as a consultant to AC Immune. W.C.M. holds a leadership position in the Trisomy 21 Research Society. He serves on committees for the Alzheimer's Project San Diego and the American Neurological Association and an NIH COBRE Grant to the University of Nebraska. W.C.M. received a royalty payment under a patent held by Stanford University licensed to Curasen. A W.C.M. laboratory member serves as PI for a grant from BioSplice, Inc. W.C.M. received the *App*‐ASO used for these studies from Ionis Pharmaceuticals Inc. H.Z. is an employee of Ionis Pharmaceuticals Inc. Author disclosures are available in the Supporting Information.

## CONSENT STATEMENT

No human subjects were included in the study. Consent was not necessary.

## Supporting information



Supporting Information

Supporting Information
