# Supplementary material for: Antisense oligonucleotides directed against App and Rab5 normalized endosomal Rab activity and reversed DS‐AD‐linked degenerative phenotypes in the Dp16 mouse model of Down syndrome
Source: Alzheimers Dement. 2025 May 7;21(5):e70022. doi: 10.1002/alz.70022 (PMC12058459; doi:10.1002/alz.70022)
Supplement: Supplementary file 1 — Supporting Information [file ALZ-21-e70022-s001.docx]

**Supplementary Figures**


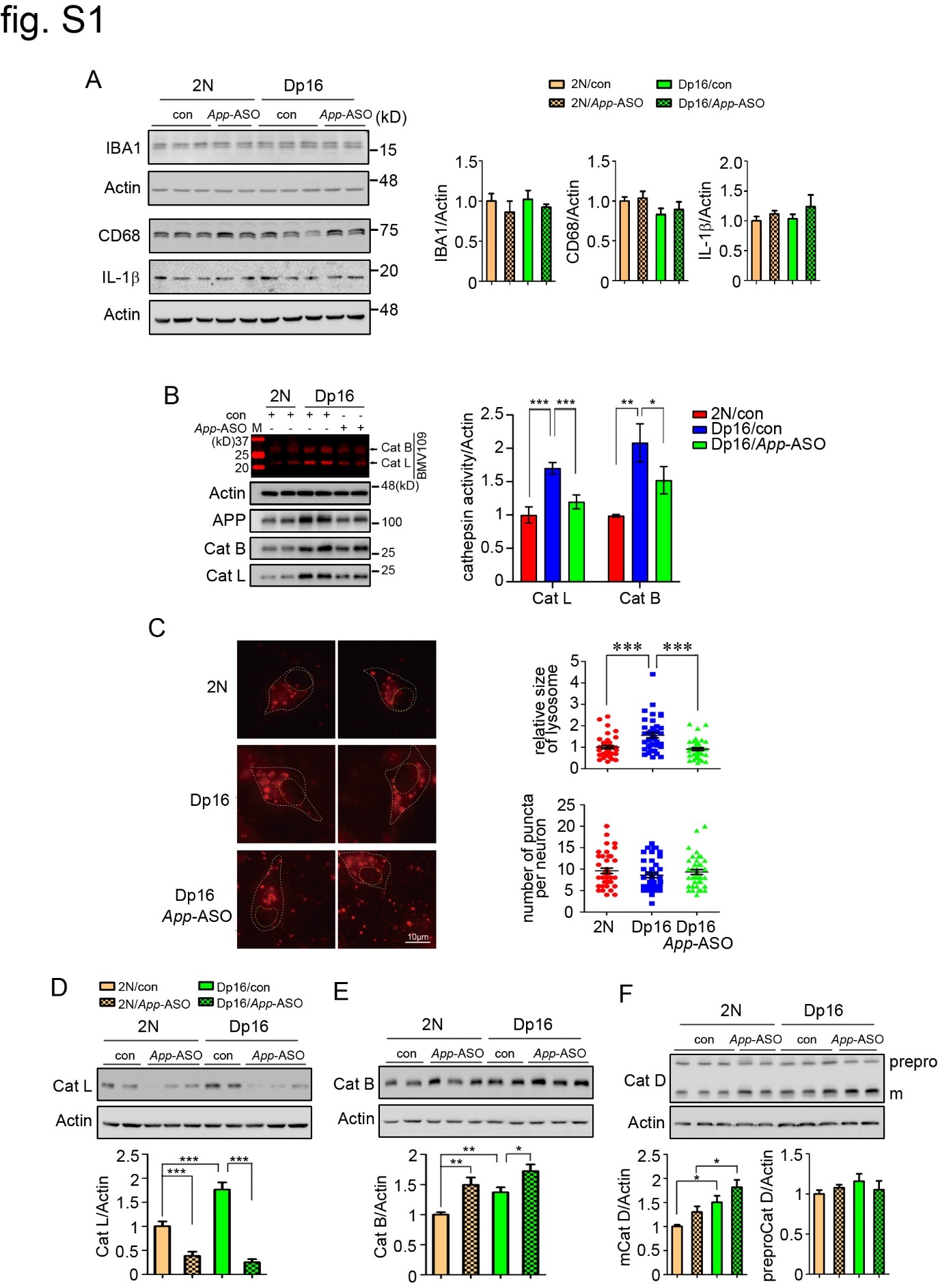


**Figure S1. The effects of *App*-ASO on the levels of cathepsins *in vivo* and *in vitro*.** (A) The effects of *App*-ASO on the levels of IBA1, CD68, and IL-1β in the cortexes of 2N and Dp16 mice were analyzed by Western blotting assay. Quantitation and statistical analysis are shown in the right panel. (B) The activities of cathepsins B and L in 2N, Dp16, and Dp16 neurons treated with *App*-ASO (100 nM, 96 hours) were assessed by fluorescent SDS-PAGE cysteine cathepsin activity profiling using BMV109. Sample fluorescence intensities were then normalized to actin. (C) Lysosome size and number were evaluated in 2N, Dp16, Dp16 neurons treated with *App*-ASO by LysoTracker. (D-F) The levels of cathepsins L, B, and D in the cortex of vehicle or *App*-ASO-treated 2N and Dp16 mice were measured. Quantitative statistical analysis was shown on the cognate lower panels. One-way ANOVA followed by Newman-Keuls Multiple Comparison Test; n = 6 for B, n = 5 mice for each group for A and D to F, n = 3 for C; **P* < 0.05, ***P* < 0.01, ****P* < 0.001.


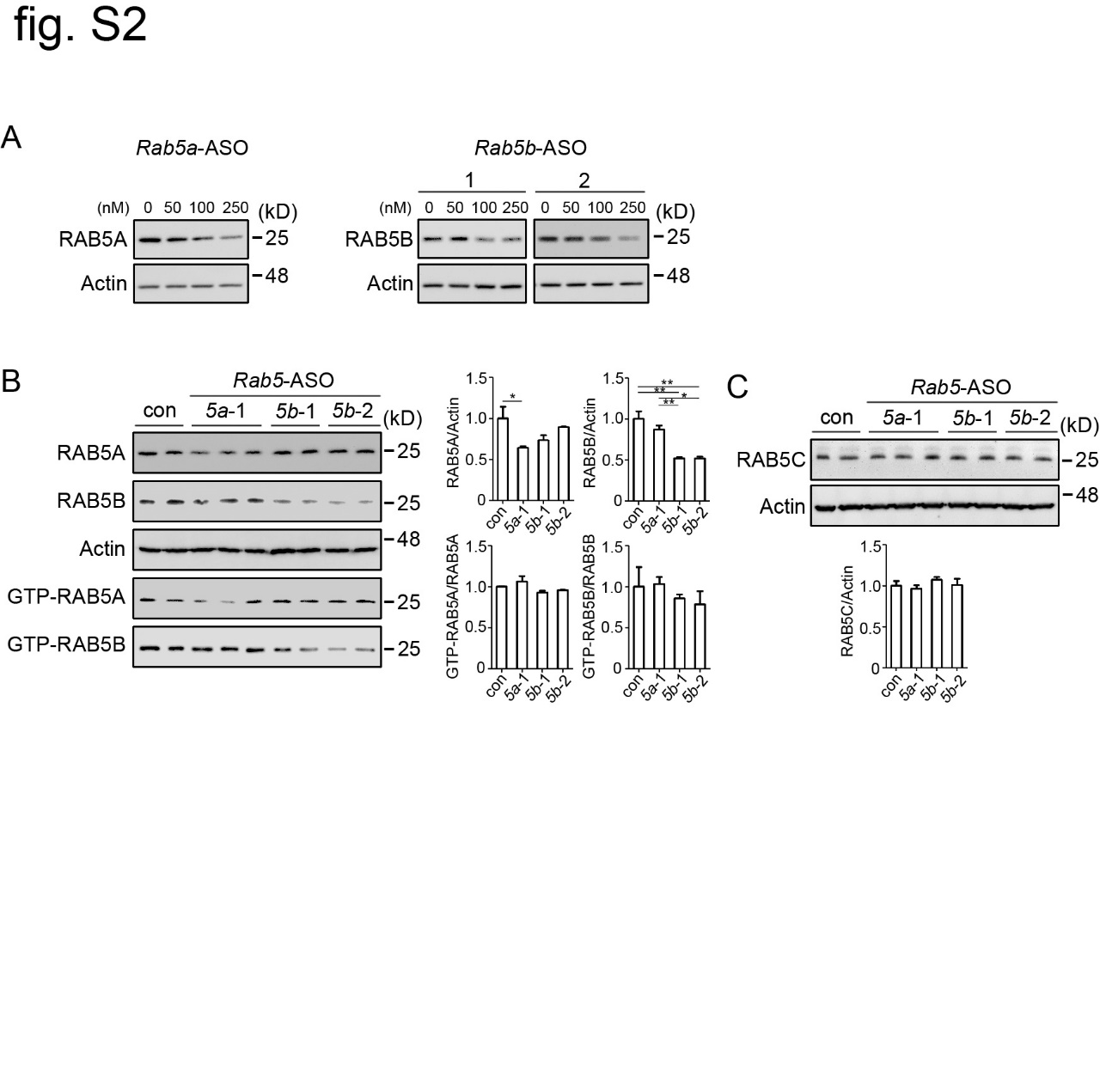


**Figure S2. Screening of *Rab5*-ASOs *in vitro* and *in vivo*.** (A) Primary cortical neurons were treated with varying concentrations of *Rab5a*-ASO or *Rab5b*-ASO at DIV5, and after 96 hours, the efficiency of these ASOs was examined by Western blotting. (B) Male 2N mice aged 3-5 months were given a single ICV injection of either *Rab5a*-ASO, *Rab5b*-ASO (100 µg), or vehicle, and this treatment was continued for two weeks. The cortex of each mouse was then dissected and processed to measure the levels of RAB5 family members using Western blotting, with β-Actin serving as a loading control. The activities of RAB5A and RAB5B were also assessed using the GTP agarose pull-down assay. (C) The levels of RAB5C were measured using the same samples as those in panel B. One-way ANOVA followed by Newman-Keuls Multiple Comparison Test; n = 2-3; **P* < 0.05, ***P* < 0.01.


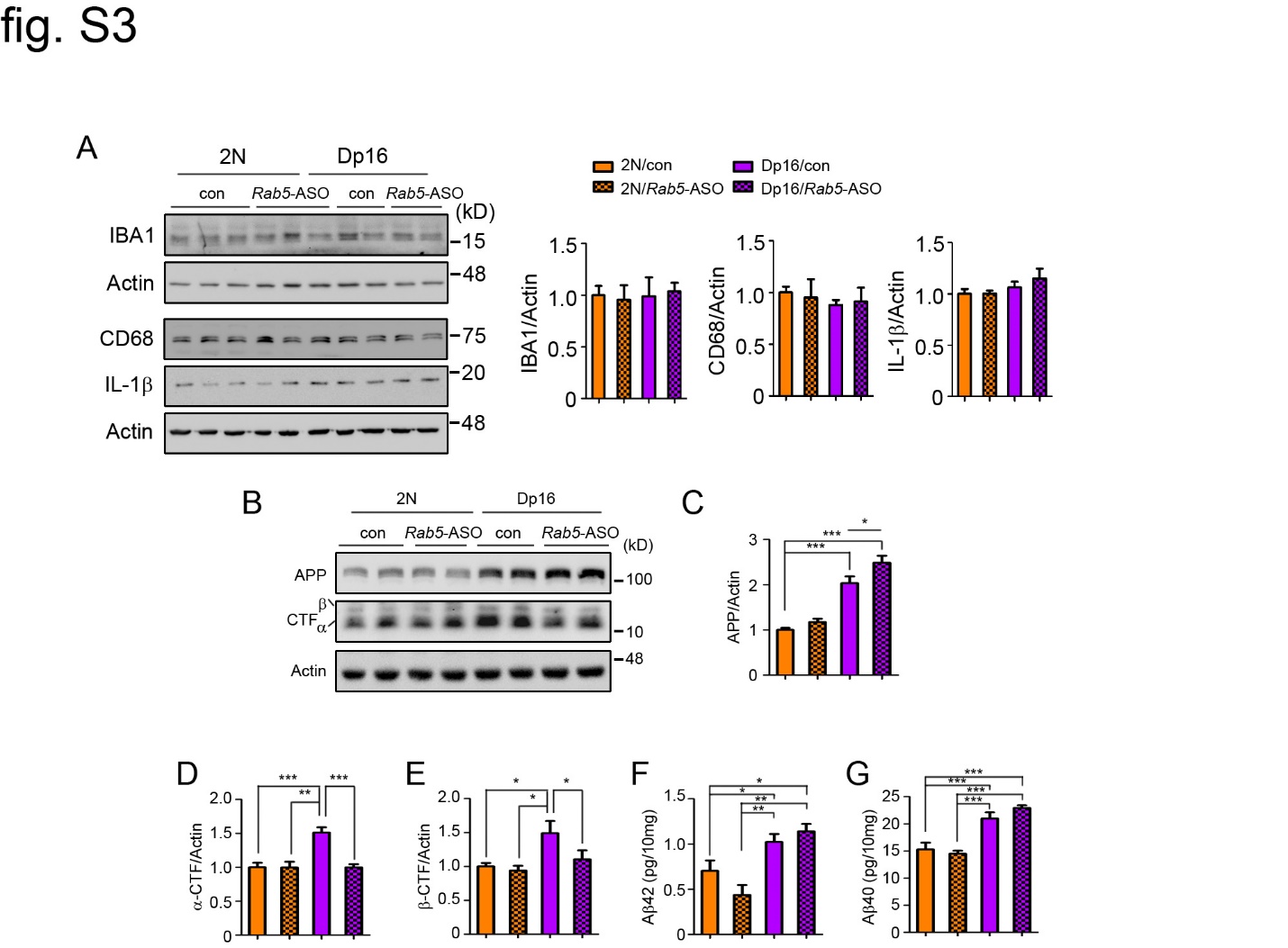


**Figure S3.** ***Rab5*-ASO reduced the levels of CTFs in Dp16 brains.** (A) The effects of *Rab5*-ASO on the levels of IBA1, CD68, and IL-1β in the cortexes of 2N and Dp16 mice were analyzed by Western blotting assay. Quantitation and statistical analysis are shown in the right panel. (B) To measure the levels of fl-APP and CTF, partial cortex samples were processed for Western blot analysis, with β-actin used as a loading control. (C-E) The statistical analysis of fl-APP, α-CTF, and β-CTF in each group was presented. (F, G) Aβ42 and Aβ40 in the cortex were measured by MSD assay. One-way ANOVA followed by Newman-Keuls Multiple Comparison Test; n = 4-5; **P* < 0.05, ***P* < 0.01, ****P* < 0.001.


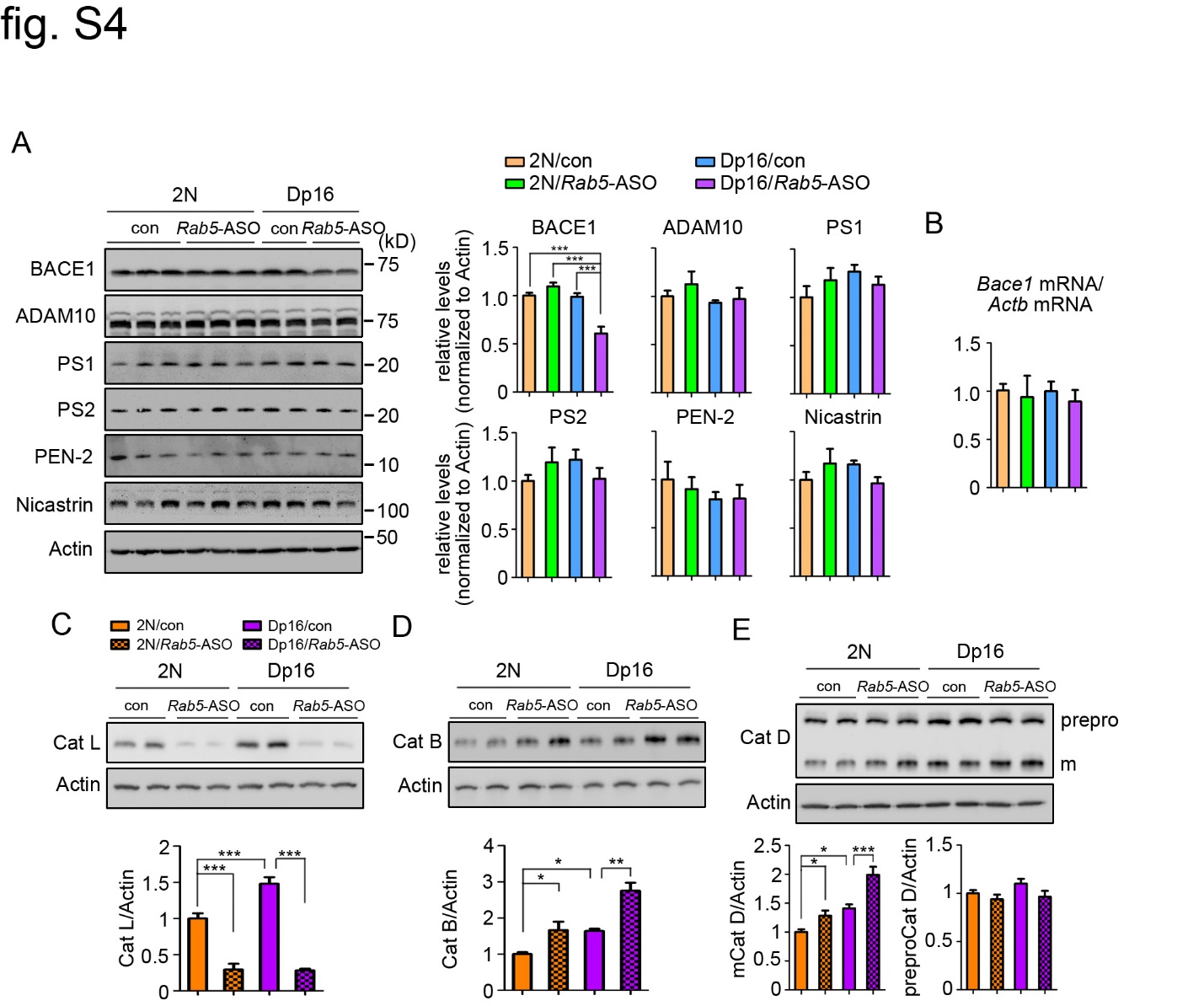


**Figure S4. The effects of *Rab5*-ASOs on the levels of APP processing enzymes and cathepsins *in vivo*.** (A) The levels of ADAM10, BECE1, and subunits of γ-secretase in the frontal cortex of vehicle or *Rab5*-ASO-treated 2N and Dp16 mice were measured and quantitative statistical analysis was shown on the right panels. (B) RT-PCR was used to analyze the mRNA levels of *Bace1* in the same mice. (C-E) The levels of cathepsins L, B, and D in the cortex of vehicle or *Rab5*-ASO-treated 2N and Dp16 mice were measured and quantitative statistical analysis was shown on the cognate lower panels. One-way ANOVA followed by Newman-Keuls Multiple Comparison Test; n = 4-5; **P* < 0.05, ***P* < 0.01, ****P* < 0.001.


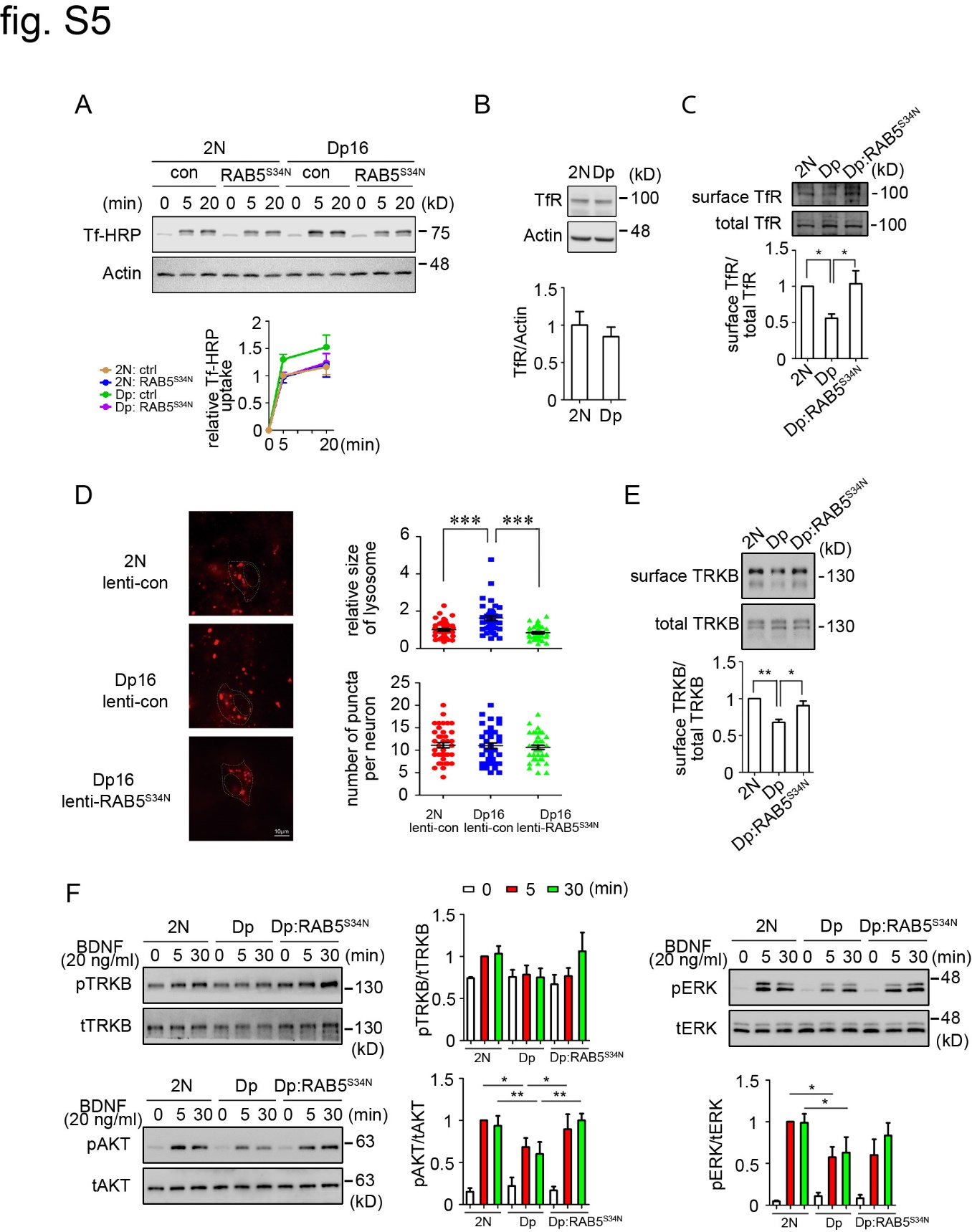


**Figure S5. RAB5 hyperactivation contributes to enhanced endocytosis and deficient BDNF signaling in primary Dp16 neurons.** (A) The endocytosis capacities of 2N and Dp16 cortical neurons infected with the RAB5^S34N^ or control lentivirus were evaluated by incubation with 10 µg/ml biotin-transferrin (Tf-biotin) for indicated durations followed by detection of internalized Tf-biotin with HRP-Conjugated Streptavidin. (B) The levels of transferrin receptor (TfR) were evaluated in the brains of 2N and Dp16 mice. (C) Surface biotinylation was applied to 2N and Dp16 cortical neurons infected with the RAB5^S34N^ or control lentivirus followed by evaluation of TfR levels on the plasma membrane with Western blotting. (D) Lysotracker was applied to 2N and Dp16 cortical neurons infected with the RAB5^S34N^ or control lentivirus to evaluate lysosome size and number. (E) Surface biotinylation was applied to 2N and Dp16 cortical neurons infected with the RAB5^S34N^ or control lentivirus followed by evaluation of TRKB levels on the plasma membrane with Western blotting. (F) 2N and Dp16 cortical neurons were starved for 2 hours in neurobasal media and stimulated with 20 ng/mL BDNF for the periods indicated followed by Western blotting to measure the BDNF-induced signaling transduction. One-way ANOVA followed by Newman-Keuls Multiple Comparison Test for C-F; n = 5 for B, n = 3 for all other panels; **P* < 0.05, ***P* < 0.01, ****P* < 0.001.

**Table S1. ASOs used in this study**

| ASO sequence | |
| --- | --- |
| *App*-ASO | G^1^sT^1^oG^1^oT^1^oA^1^sdTsdTsdTsdTsdTsdTsdTsdC^2^sdTsdGsA^1^oC^1,2^oC^1,2^sC^1,2^sT^1^  1=2’-MOE; 2=5Me; s=PS; o=PO; d=deoxy |
| *Rab5a*-ASO | A^1^sT^1^sT^1^sC^1,2^sC^1,2^sdAsdAsdC^2^sdC^2^sdTsdGsdAsdGsdC^2^sdAsC^1,2^sC^1,2^sT^1^sC^1,2^sA^1^  1=2’-MOE; 2=5Me; s=PS; d=deoxy |
| *Rab5b-*ASO-1 | T^1^sT^1^sA^1^sA^1^sA^1^sdGsdTsdGsdC^2^sdAsdAsdAsdC^2^sdTsdGsC^1,2^sA^1^sG^1^sG^1^sG^1^  1=2’-MOE; 2=5Me; s=PS; d=deoxy |
| *Rab5b*-ASO-2 | G^1^sT^1^sT^1^sT^1^sC^1,2^sdTsdC^2^sdC^2^sdC^2^sdAsdAsdAsdC^2^sdTsdC^2^sT^1^sG^1^sG^1^sC^1,2^sT^1^  1=2’-MOE; 2=5Me; s=PS; d=deoxy |

**Table S2. DNA primers used in this study**

| Primers for RT-PCR | | |
| --- | --- | --- |
| Primer name | Forward primer (5’-3’) | Reverse primer (5’-3’) |
| Mouse *Rab5a* | CAAGAACGGTATCATAGCTTAGCAC | CTTGCCTTTGAAGTTCTTTAACCC |
| Mouse *Rab5b* | GCAGGGAACAAAGCTGACCT | CTGGGGTTCGCTCTTTGG |
| Mouse *Rab7* | ATGGTGGACGACAGACTTGT | CAACAAAAGGGAAGTTCTCGG |
| Mouse *App* | TGCTGAAGATGTGGGTTCGA | GACAATCACGGTTGCTATGACAA |
| Mouse *Bace1* | GGAACCCATCTCGGCATCC | TCCGATTCCTCGTCGGTCTC |
| Mouse *Actb* | GATCATTGCTCCTCCTGAGC | ACATCTGCTGGAAGGTGGAC |
| Mouse *Gapdh* | TCCACCACCCTGTTGCTGTA | ACCACAGTCCATGCCATCAC |

**Table S3. Antibodies used in this study**

| **Antibody** | **Vendor** | **Identifier** |
| --- | --- | --- |
| Rabbit polyclonal anti-RAB5B | Santa Cruz Biotechnology | Cat# sc-598; RRID: AB_2175453 |
| Rabbit monoclonal anti-RAB7 | Cell Signaling Technology | Cat# 9367; RRID: AB_1904103 |
| Rabbit monoclonal anti-RAB11A | Abcam | Cat# ab128913; RRID: AB_11140633 |
| Rabbit monoclonal anti-APP/CTF | Abcam | Cat# ab32136; RRID: AB_2289606 |
| Mouse monoclonal anti-β-Actin | Proteintech | Cat# 60008-1-Ig; RRID: AB_2289225 |
| Mouse monoclonal anti-RAB5A | Synaptic Systems | Cat# 108111; RRID: AB_2619777 |
| Rabbit polyclonal anti-RAB5C | Abclonal | Cat# A7342; RRID: AB_2767879 |
| Mouse monoclonal anti-CCZ1 | Santa Cruz Biotechnology | Cat# sc-514290 |
| Rabbit polyclonal anti-SH3BP5 | GeneTex | Cat# GTX112002; RRID: AB_11166818 |
| Rabbit polyclonal anti-APLP1 | Calbiochem | Cat# 171615; RRID: AB_10683250 |
| Rabbit polyclonal anti-APLP2 | Calbiochem | Cat# 171617; RRID: AB_565357 |
| Mouse monoclonal anti-BACE1 | Novus Biologicals | Cat# MAB931; RRID: AB_2258772 |
| Rabbit polyclonal anti-ADAM10 | Abcam | Cat# ab1997; RRID: AB_302747 |
| Rabbit monoclonal anti-Presenilin 1 (PS1) | Cell Signaling Technology | Cat# 5643; RRID: AB_10706356 |
| Rabbit monoclonal anti-Presenilin 2 (PS2) | Cell Signaling Technology | Cat# 9979; RRID: AB_10829910 |
| Rabbit monoclonal anti-PEN-2 | Cell Signaling Technology | Cat# 8502; RRID: AB_11130041 |
| Rabbit monoclonal anti-Nicastrin | Cell Signaling Technology | Cat# 5665; RRID: AB_10694688 |
| Mouse monoclonal anti-CD71 (Transferrin receptor) | Thermo Fisher Scientific | Cat# 13-6800; RRID: AB_2533029 |
| Mouse monoclonal anti-TRKB | BD Biosciences | Cat# 610101; RRID: AB_397507 |
| Rabbit polyclonal anti-pTRKB (Tyr490) | Dr. Moses Chao (New York University) | N/A |
| Rabbit monoclonal anti-pAKT (Ser473) | Cell Signaling Technologies | Cat# 4060; RRID: AB_2315049 |
| Mouse monoclonal anti-AKT | Cell Signaling Technologies | Cat# 2920; RRID: AB_1147620 |
| Rabbit monoclonal anti-pERK1/2 (Thr202/Tyr204) | Cell Signaling Technologies | Cat# 4370; RRID: AB_2315112 |
| Rabbit monoclonal anti-ERK1/2 | Cell Signaling Technologies | Cat# 4695; RRID: AB_390779 |
| Rabbit monoclonal anti-pCREB (Ser133) | Cell Signaling Technologies | Cat# 9198; RRID: AB_2561044 |
| Rabbit monoclonal anti-CREB | Cell Signaling Technologies | Cat# 9197; RRID: AB_331277 |
| Mouse monoclonal anti-Syntaxin 1A | MilliporeSigma | Cat# S0664; RRID: AB_477483 |
| Rabbit polyclonal anti-SNAP25 | Proteintech | Cat# 14903-1-AP; RRID: AB_2192051 |
| Mouse monoclonal anti-PHF1 | Dr. Peter Davies (Albert Einstein University) | Cat# PHF1; RRID: AB_2315150 |
| Rabbit polyclonal anti-pTAU (Thr205) | Thermo Fisher Scientific | Cat# OPA1-03153; RRID: AB_326050 |
| Mouse monoclonal anti-TAU (TAU-5) | Thermo Fisher Scientific | Cat# AHB0042; RRID: AB_2536235 |
| Rabbit monoclonal anti-pGSK3β (Ser9) | Cell Signaling Technologies | Cat# 5558; RRID: AB_10013750 |
| Rabbit monoclonal anti-GSK3β | Cell Signaling Technologies | Cat# 12456; RRID: AB_2636978 |
| Rabbit anti-ASO backbone | Ionis Pharmaceuticals Inc | N/A |
| Rabbit monoclonal anti-cathepsin B | Cell Signaling Technologies | Cat# 31718; RRID: AB_2687580 |
| Mouse monoclonal anti-cathepsin L (mouse) | Santa Cruz Biotechnology | Cat# sc-390367; RRID: AB_2827873 |
| Rabbit polyclonal anti-cathepsin D | Cell Signaling Technologies | Cat# 69854 |
| Rabbit polyclonal anti-IBA1 | Proteintech | Cat# 10904-1-AP; RRID: AB_2224377 |
| Rat monoclonal anti-CD68 | Thermo Fisher Scientific | Cat# 14-0681-82; RRID: AB_2572857 |
| Mouse monoclonal anti-IL-1β | Cell Signaling Technologies | Cat# 12242; RRID: RRID:AB_2715503 |
| Goat anti-rabbit IgG-HRP | Jackson ImmunoResearch Laboratories | Cat# 111-035-144; RRID: AB_2307391 |
| Goat anti-mouse IgG-HRP | Jackson ImmunoResearch Laboratories | Cat# 115-035-003; RRID: AB_10015289 |
| Goat anti-rat IgG-HRP | Jackson ImmunoResearch Laboratories | Cat#: 112-035-003; RRID: AB_2338128 |
| Donkey anti-rabbit IgG Alexa Fluor™ 555 | Thermo Fisher Scientific | Cat# A-31572; RRID: AB_162543 |
